# Anion exchanger 2 suppresses cellular movement and has prognostic significance in esophageal squamous cell carcinoma

**DOI:** 10.18632/oncotarget.25417

**Published:** 2018-05-25

**Authors:** Atsushi Shiozaki, Shoichiro Hikami, Daisuke Ichikawa, Toshiyuki Kosuga, Hiroki Shimizu, Michihiro Kudou, Yuzo Yamazato, Toshiyuki Kobayashi, Katsutoshi Shoda, Tomohiro Arita, Hirotaka Konishi, Shuhei Komatsu, Takeshi Kubota, Hitoshi Fujiwara, Kazuma Okamoto, Mitsuo Kishimoto, Eiichi Konishi, Yoshinori Marunaka, Eigo Otsuji

**Affiliations:** ^1^ Division of Digestive Surgery, Department of Surgery, Kyoto Prefectural University of Medicine, Kyoto 602-8566, Japan; ^2^ Department of Gastrointestinal, Breast and Endocrine Surgery, Faculty of Medicine, University of Yamanashi, Chuo 409-3898, Japan; ^3^ Department of Pathology, Kyoto Prefectural University of Medicine, Kyoto 602-8566, Japan; ^4^ Departments of Molecular Cell Physiology and Bio-Ionomics, Graduate School of Medical Science, Kyoto Prefectural University of Medicine, Kyoto 602-8566, Japan; ^5^ Japan Institute for Food Education and Health, St. Agnes' University, Kyoto 602-8013, Japan

**Keywords:** AE2, esophageal squamous cell carcinoma, MMPs, migration, cellular physiology

## Abstract

**Background:**

Recent studies have reported essential roles for various intracellular pH regulators in epithelial carcinogenesis and tumor progression. The aims of the present study were to investigate the role of anion exchanger 2 (AE2) in the regulation of tumor progression-related genes and the prognostic value of its expression in esophageal squamous cell carcinoma (ESCC).

**Results:**

AE2 was strongly expressed in KYSE170 and TE13 cells. The depletion of AE2 in these cells increased cell migration and inhibited the induction of apoptosis. The results of the microarray analysis revealed that various matrix metalloproteinase (MMP) signaling pathway-related genes, such as MMP1, MMP12, and TIMP4, were up- or down-regulated in AE2-depleted KYSE170 cells. Immunohistochemical staining showed that AE2 was primarily located in the cell membranes or cytoplasm of carcinoma cells, and its expression pattern at the invasive front of the tumor was related to the pT category. Prognostic analyses revealed that the low-grade expression of AE2 at the invasive front was associated with shorter postoperative survival.

**Conclusions:**

The results of the present study suggest that reductions in AE2 in ESCC enhance cellular movement by activating MMP signaling pathways and are related to a poor prognosis in patients with ESCC.

**Methods:**

In human ESCC cell lines, knockdown experiments were conducted using AE2 siRNA, and the effects on cellular movement and survival were analyzed. The gene expression profiles of cells were examined using a microarray analysis. An immunohistochemical analysis was performed on 61 primary tumor samples obtained from ESCC patients who underwent esophagectomy.

## INTRODUCTION

Recent studies indicated that cytoplasmic pH regulated by ion transporters plays essential roles in various fundamental mechanism of tumor cells [1–4]. Tumor cells have an acidic extracellular microenvironment which is absolutely different from that in normal tissues [5–7]. Accumulating evidences have indicated that an acidic extracellular pH (pHe) is linked to an alkaline intracellular pH (pHi) that controls the malignant phenotype of cancer cells. The development and maintenance of such a microenvironment are directly driven by pH regulators, such as the anion exchanger (AE), Na^+^/H^+^ exchanger (NHE), monocarboxylate transporter (MCT), vacuolar H+-ATPase, and carbonic anhydrase (CA) [5–7]. We previously investigated the functions of ion transporters [8, 9], water channels [10], and pHi regulators including AE1 [11], NHE1 [12], and CA12 [13] in esophageal squamous cell carcinoma (ESCC). Our findings suggested that these cellular physiological factors are effective biomarkers, and their regulation may produce novel strategies for future therapies for ESCC [4].

The AE is a membrane protein that mediates the exchange of chloride (Cl^−^) with bicarbonate (HCO_3_^−^) across the plasma membrane, and contributes to a decrease in pHi, the regulation of intracellular Cl^−^ concentrations, bicarbonate metabolism, and cellular volume [14–17]. Three isoforms of the AE have been identified to date; AE2 is ubiquitously expressed in most tissues, whereas AE1 is specifically expressed in erythrocytes and kidney, and the expression of AE3 has been detected in the brain, retina, and heart [14–17]. Recent studies revealed that AE2 plays critical roles in various cancers such as gastric cancer [18–20], hepatocellular carcinoma [21–23], colon cancer [24], cervical cancer [25], and renal carcinoma [26]. However, the roles of AE2 in tumor progression in patients with ESCC and the clinical significance of its expression remain unclear.

Therefore, the aims of the present study were to clarify the roles of AE2 in the regulation of genes involved in tumor progression and the clinicopathological significance of its expression in ESCC. A microarray analysis showed that the depletion of AE2 with siRNA changed the expression levels of many genes involved in cellular movement. We also analyzed the expression of AE2 in human ESCC samples and investigated its relationships with the clinicopathological features and prognosis of ESCC patients. Our results indicate that AE2 plays a crucial role in tumor progression in ESCC patients.

## RESULTS

### Protein expression of AE2 in ESCC cells

**Figure 1:**
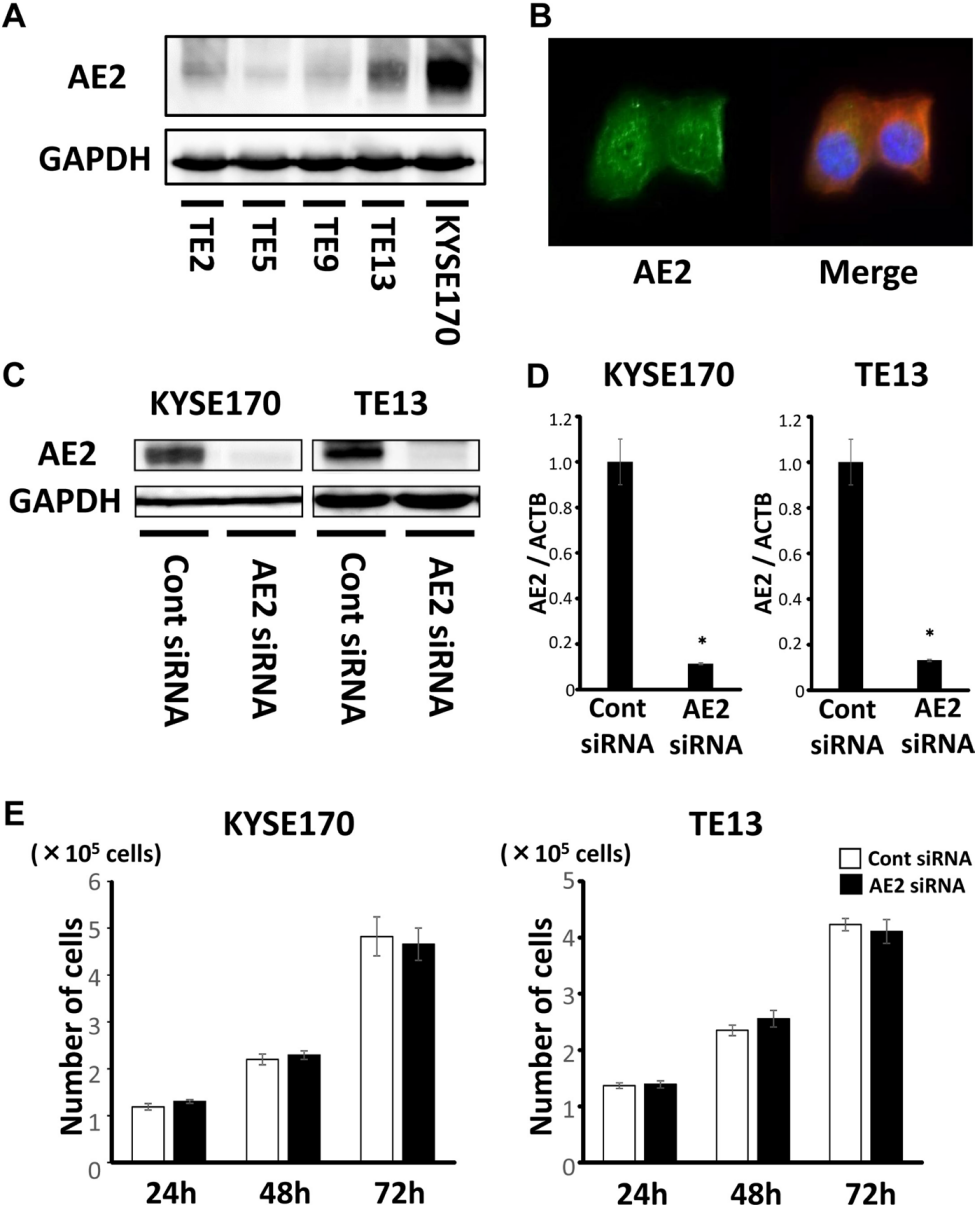
Expression of AE2 in ESCC cells (**A**) AE2 protein expression was analyzed in 5 ESCC cell lines. Western blotting showed that AE2 was strongly expressed in KYSE170 and TE13 cells. (**B**) The subcellular distribution of the AE2 protein was assessed by confocal microscopy. Immunofluorescent staining with the AE2 antibody demonstrated that AE2 was mainly distributed in the cell membranes or cytoplasm of KYSE170 cells. (**C**) Western blotting revealed that AE2 siRNA effectively reduced AE2 protein levels in KYSE170 and TE13 cells. (**D**) AE2 siRNA effectively reduced AE2 mRNA levels in KYSE170 and TE13 cells. Mean ± SEM. *n* = 3. ^*^p < 0.05 (significantly different from control siRNA). (**E**) The down-regulation of AE2 did not change the proliferation of KYSE170 or TE13 cells. The number of cells was counted 24, 48, and 72 h after siRNA transfection. Mean ± SEM. *n* = 4. ^*^p < 0.05 (significantly different from control siRNA).

In order to clarify the role of AE2 in ESCC, we examined 5 ESCC cell lines: TE2, TE5, TE9, TE13, and KYSE170, for AE2 protein expression. Western blotting of these cell lines showed that AE2 was strongly expressed in KYSE170 and TE13 cells (Figure 1A). The subcellular distribution of the AE2 protein in KYSE170 cells was analyzed with confocal microscopy. Immunofluorescent staining with the AE2 antibody demonstrated that AE2 was distributed in the cell membranes or cytoplasm of KYSE170 cells (Figure 1B).

We conducted knockdown experiments using AE2 siRNA in KYSE170 and TE13 cells and investigated the effects of AE2 depletion on cell growth. AE2 siRNA effectively reduced AE2 protein levels (Figure 1C) and AE2 mRNA levels (Figure 1D) in both cell lines. In KYSE170 and TE13 cells, the cell counts of AE2-depleted cells were not significantly different from those of control siRNA-transfected cells at 24, 48, and 72 h after siRNA transfection (Figure 1E). Even if the incubation time after siRNA transfection was extended more, the same result was obtained (Supplementary Figure 1). We also conducted overexpression study. Cells transfected Control-HaloTag^®^ plasmid and AE2-HaloTag^®^ plasmid were stained in red (Supplementary Figure 2A), and AE2 plasmid increased AE2 mRNA levels (Supplementary Figure 2B). AE2 overexpression in KYSE170 cells decreased cell growth (Supplementary Figure 3A). AE2 overexpression partially reduced cell cycle progression from the G1 to S phase in KYSE170 cells (Supplementary Figure 3B). Further, to determine the role of AE2 in tumor growth *in vivo*, a xenograft model in nude mice was used. Injection of AE2 plasmid transfected KYSE170 cells subcutaneously into nude mice resulted in significantly smaller tumor volumes than those of the control plasmid transfected cells (Supplementary Figure 4A, 4B). The average weight of AE2 plasmid transfected tumors was reduced in comparison with control tumors (Supplementary Figure 4C).

### AE2 controls the survival of ESCC cells

**Figure 2:**
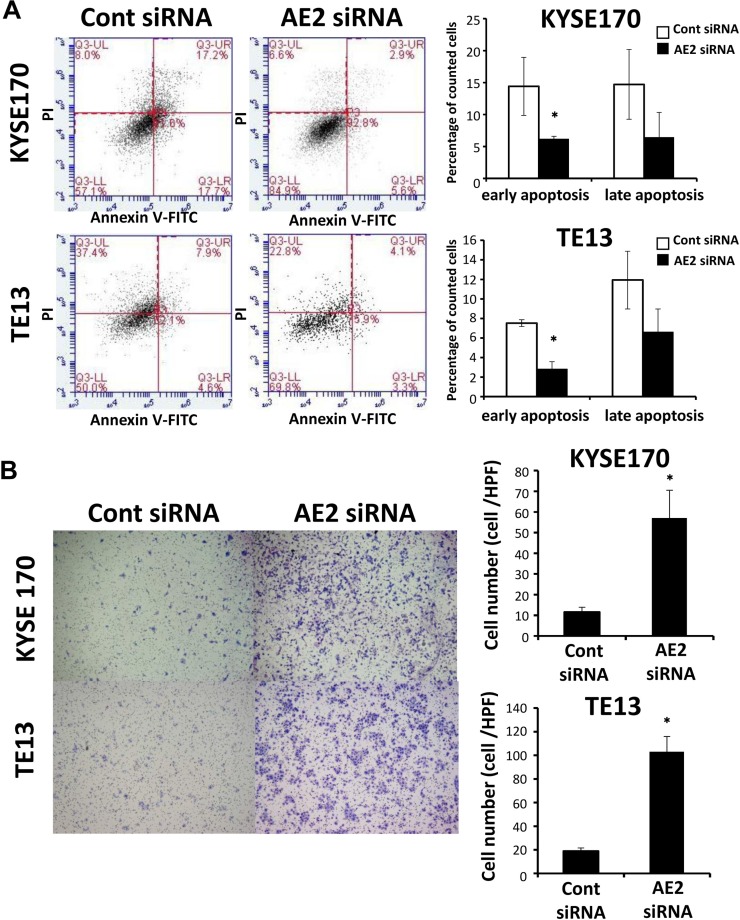
AE2 controls the survival and migration of ESCC cells (**A**) The down-regulation of AE2 significantly decreased staurosporine stimulus-induced early apoptosis in KYSE170 and TE13 cells. Cells transfected with control or AE2 siRNA were treated with staurosporine (200 nmol/L) for 24 h. Apoptosis was assessed by flow cytometry using PI/annexin V double staining. Mean ± SEM. *n* = 3. ^*^p < 0.05 (significantly different from control siRNA). (**B**) The down-regulation of AE2 increased the migration of KYSE170 and TE13 cells. Cell migration was examined using the Boyden chamber assay. Mean ± SEM. *n* = 3. ^*^p < 0.05 (significantly different from control siRNA).

In order to investigate the role of AE2 in ESCC cell survival, we treated KYSE170 and TE13 cells with AE2 siRNA and analyzed apoptosis. The down-regulation of AE2 significantly decreased staurosporine stimulus-induced early apoptosis (annexin V positive/PI negative) in KYSE170 and TE13 cells (Figure 2A). These results indicate that AE2 expression influences the survival of ESCC cells.

### AE2 controls cellular movement in ESCC cells

We conducted knockdown experiments with AE2 siRNA in ESCC cells, and analyzed the effects of the knockdown of AE2 on cell migration and invasion using the Boyden chamber assay. In KYSE170 and TE13 cells, AE2 siRNA significantly increased cell migration (Figure 2B). Furthermore, the down-regulation of AE2 significantly increased cell invasion in KYSE170 cells (Supplementary Figure 5A). In the wound healing assay, the down-regulation of AE2 significantly increased wound closure in TE13 cells (Supplementary Figure 5B). AE2 overexpression in KYSE170 cells decreased cell migration (Supplementary Figure 6) as opposed to knockdown of AE2. These results suggest that AE2 plays an important role in regulating the movement of ESCC cells.

### Gene expression profiles of AE2-depleted cells

We analyzed the gene expression profiles of AE2-depleted KYSE170 cells in microarray and bioinformatics studies. The results of the microarray analysis showed that the expression levels of 1811 genes displayed fold changes of >2.0 in KYSE170 cells upon the depletion of AE2. Among these genes, 544 were up-regulated and 1267 were down-regulated in AE2 siRNA-depleted KYSE170 cells. AE2 expression was down-regulated in AE2-depleted KYSE170 cells (fold change: −10.99). A list of 20 genes with expression levels that were the most strongly up- or down-regulated in AE2-depleted KYSE170 cells is shown in Supplementary Table 1. IPA showed that “Cancer” was one of the top-ranking diseases. Furthermore, “Cellular Movement” was the top-ranking biological function related to AE2 depletion (Supplementary Table 2), and was consistent with the results obtained in our *in vitro* studies.

### Molecular mechanisms regulated by AE2 in ESCC cells

**Figure 3:**
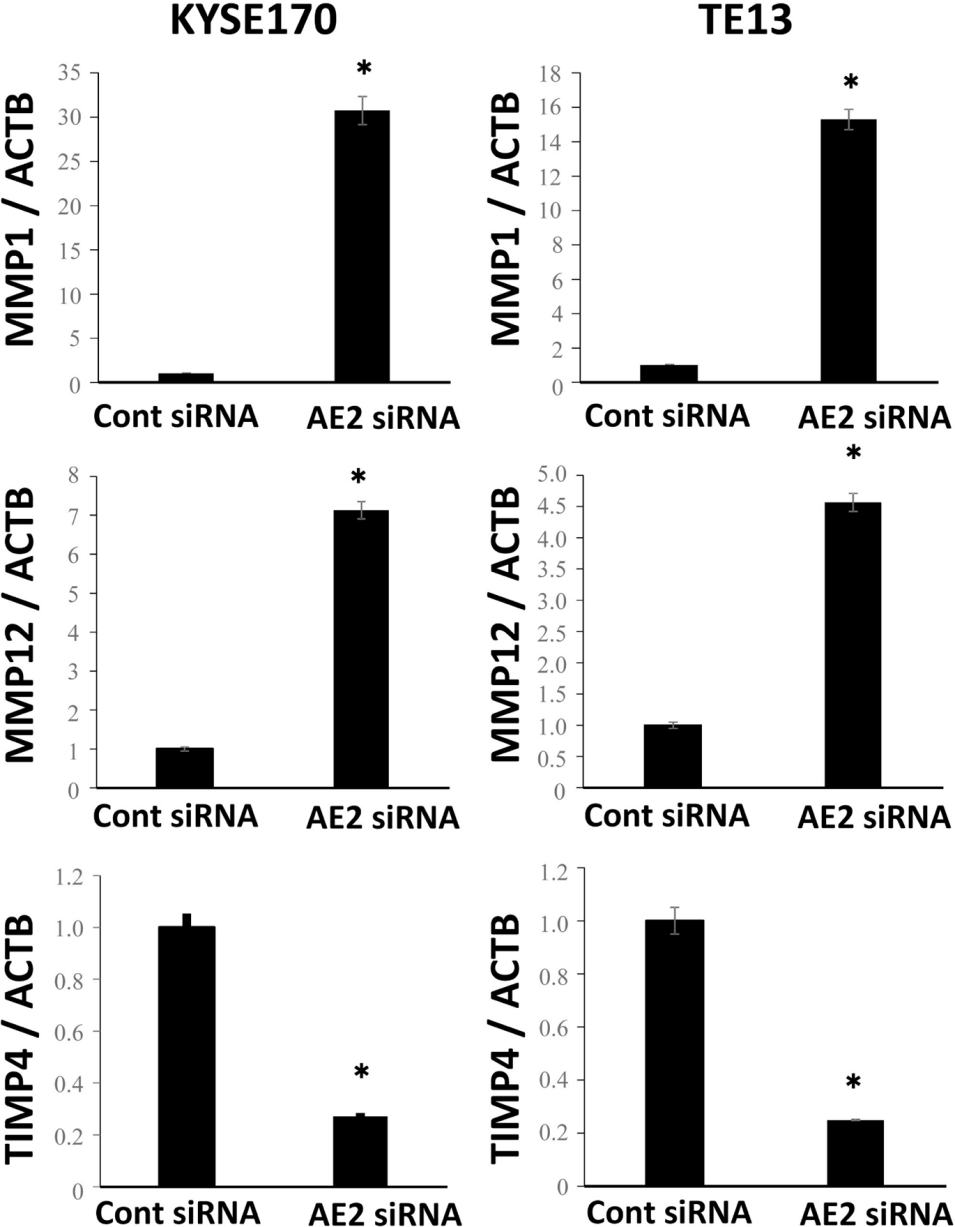
Verification of gene expression by real-time quantitative RT-PCR The expression levels of three selected MMP signaling pathway-related genes (MMP1, MMP12, and TIMP4) in AE2-depleted KYSE170 and TE13 cells were compared to those in control siRNA-transfected cells using real-time quantitative RT-PCR. Mean ± SEM. *n* = 3. ^*^p < 0.05 (significantly different from control siRNA).

We then examined the signal transduction networks induced by AE2 depletion using IPA. Regarding “Cellular Movement”, the map of “matrix metalloproteinase (MMP)” revealed that various MMP-related genes were up- or down-regulated by the depletion of AE2 (Table 1, Supplementary Figure 7). In order to confirm the results of the gene expression profiles of AE2-depleted cells, the gene expression of MMP1, MMP12, and TIMP4 was examined further using quantitative RT-PCR. The expression levels of MMP1 and MMP12 mRNA were higher in AE2-depleted KYSE170 cells than in control siRNA-transfected cells (Figure 3). The expression level of TIMP4 mRNA was lower in AE2-depleted KYSE170 cells than in control siRNA-transfected cells (Figure 3). Similar results were obtained in the TE13 cell line (Figure 3). These results were consistent with those of the microarray analysis and suggest that MMP regulation is a key mechanism by which AE2 controls the movement of ESCC cells.

**Table 1:** MMP signaling pathway-related genes with expression levels in KYSE170 cells that were changed by the depletion of AE2

MMP signaling pathway			
Symbol	Gene Name	Agilent ID	Exp Fold Change
MMP1	matrix metallopeptidase 1	A_23_P1691	4.308
MMP12	matrix metallopeptidase 12	A_23_P150316	2.734
MMP10	matrix metallopeptidase 10	A_23_P13094	2.677
MMP13	matrix metallopeptidase 13	A_33_P3221203	1.375
TIMP1	TIMP metallopeptidase inhibitor 1	A_23_P62115	–0.187
MMP11	matrix metallopeptidase 11	A_23_P57417	–1.155
TIMP3	TIMP metallopeptidase inhibitor 3	A_23_P399078	–1.439
TIMP4	TIMP metallopeptidase inhibitor 4	A_32_P70315	–3.665

### AE2 protein expression in human ESCCs

**Figure 4:**
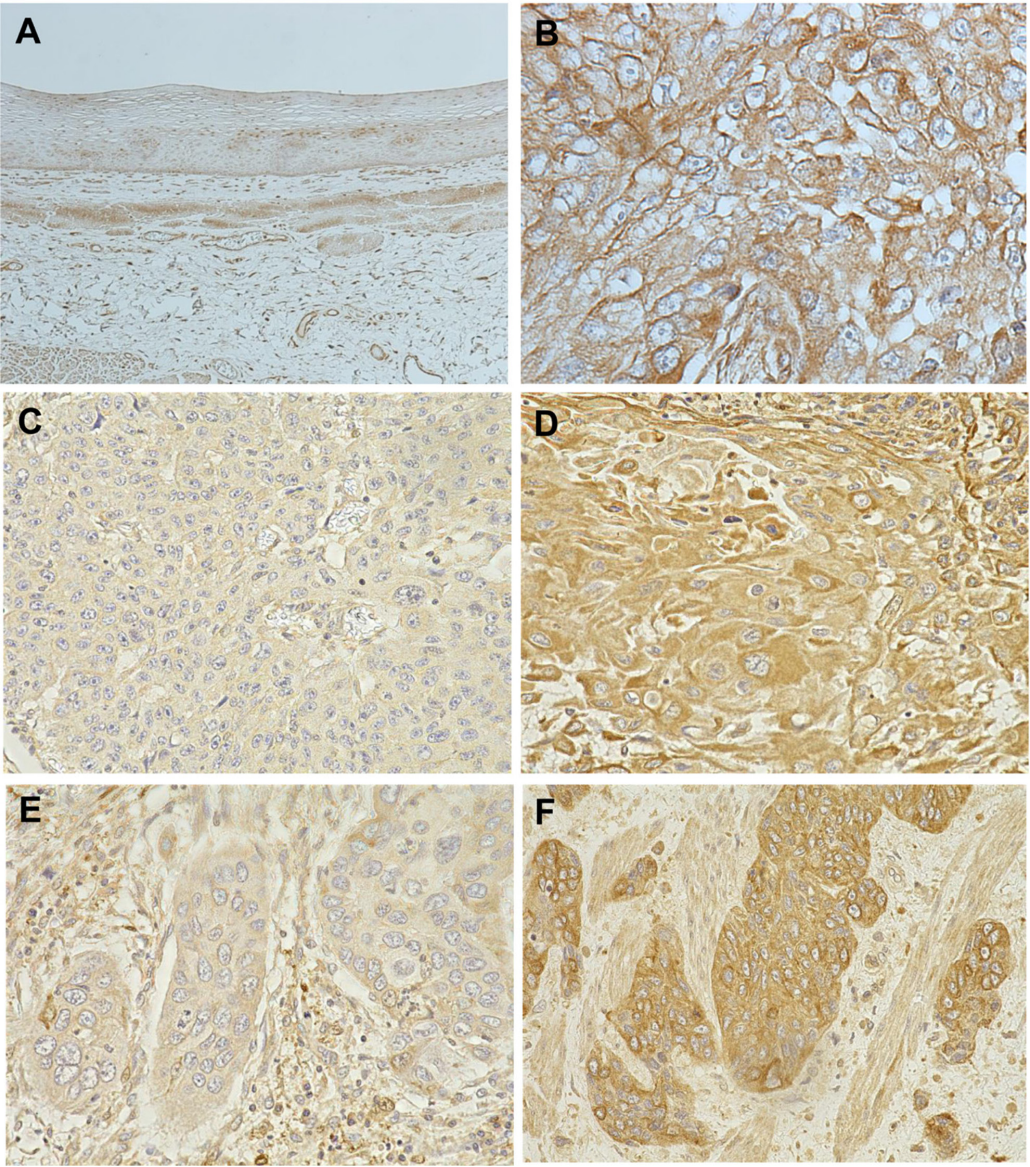
AE2 protein expression in human ESCC (**A**) Immunohistochemical staining of non-cancerous esophageal epithelia with the AE2 antibody. Magnification: ×40. (**B**) Immunohistochemical staining of primary human ESCC samples with the AE2 antibody. Magnification: ×400. (**C**) Immunohistochemical staining of primary human ESCC samples with the low-grade expression of AE2 in the whole tumor (WT). Magnification: ×400. (**D**) Immunohistochemical staining of primary human ESCC samples with the high-grade expression of AE2 in WT. Magnification: ×400. (**E**) Immunohistochemical staining of primary human ESCC samples with the low-grade expression of AE2 at the invasive front of the tumor (IF). Magnification: ×400. (**F**) Immunohistochemical staining of primary human ESCC samples with the high-grade expression of AE2 at the IF. Magnification: ×400.

An immunohistochemical investigation with the AE2 antibody revealed the expression of AE2 in the parabasal cell layer of the normal esophageal mucosa (Figure 4A). We examined the expression of AE2 in 61 primary tumor samples of human ESCC based on their immunohistochemical reactivity. The AE2 protein was mostly expressed in the cell membranes or cytoplasm of carcinoma cells (Figure 4B). Referentially, we also show the sample which includes both normal esophageal epithelia and ESCC cells (Supplementary Figure 8). The AE2 score in the whole tumor (WT) varied widely between the tumors. The minimum AE2 score was 0.15, while the maximum was 2.7 (median = 1.4; mean ± standard deviation (SD) = 1.32 ± 0.574). Regarding the expression of AE2 in WT, we divided ESCC patients into 2 groups based on mean staining scores; a low-grade AE2 expression group with staining scores <1.32, *n* = 30, and a high-grade AE2 expression group with staining scores ≥1.32, *n* = 31 (Figure 4C, 4D, Supplementary Figure 9A, 9B). The relationships between the expression of AE2 in WT and various clinicopathological parameters were analyzed (Table 2). A correlation was not found between the comparison of AE2 scores in WT and clinicopathological features (Table 2).

**Table 2:** Relationships between clinicopathological features of ESCC and the expression of AE2

Variable	AE2 staining score in WT		*p* value	AE2 staining score at the IF		*p* value
	High	Low		High	Low	
	(*n* = 31)	(*n* = 30)		(*n* = 35)	(*n* = 26)	
Gender						
Male	25	27	0.4729	27	25	0.0653
Female	6	3		8	1	
Age						
<65 years	16	21	0.1919	21	16	1.0000
≥65 years	15	9		14	10	
Tumor size						
<50 mm	22	18	0.4263	24	16	0.5963
≥50 mm	9	12		11	10	
Histological type						
Well-/moderately differentiated SCC	21	23	0.5700	23	21	0.2536
Poorly differentiated SCC	10	7		12	5	
Lymphatic invasion						
Negative	11	17	0.1261	14	14	0.3107
Positive	20	13		21	12	
Venous invasion						
Negative	16	20	0.3004	18	18	01952
Positive	15	10		17	8	
pT						
pT1	17	10	0.1236	20	7	0.0221^*^
pT2-3	14	20		15	19	
pN						
pN0	14	13	1.0000	16	11	1.0000
pN1-3	17	17		19	15	
pStage						
pStage 0–I	9	8	1.0000	10	7	1.0000
pStage II–IV	22	22		25	19	

SCC: squamous cell carcinoma; pT: pathological T stage; pN: pathological N stage; pStage: pathological stage.

^*^p < 0.05: Fisher's exact test.

We then focused on the expression of AE2 at the invasive front (IF) of the tumor of ESCC (Figure 4E, 4F, Supplementary Figure 9C, 9D) and analyzed the AE2 score at the IF. The minimum AE2 score was 0, while the maximum was 2.7 at the IF (median = 1.5; mean ± SD = 1.43 ± 0.718). The AE2 score at the IF positively correlated with the AE2 score in WT (R^2^ = 0.5319, *p* < 0.0001) (Supplementary Figure 10). Regarding the expression of AE2 at the IF, we divided ESCC patients into 2 groups using the mean staining score: a low-grade AE2 expression group with staining scores <1.43, *n* = 26, and a high-grade AE2 expression group with staining scores ≥1.43, *n* = 35, and compared their clinicopathological features (Table 2). A correlation was found between the expression of AE2 at the IF and the pT category (Table 2).

### Prognostic impact of AE2 protein expression for patients with ESCC

**Figure 5:**
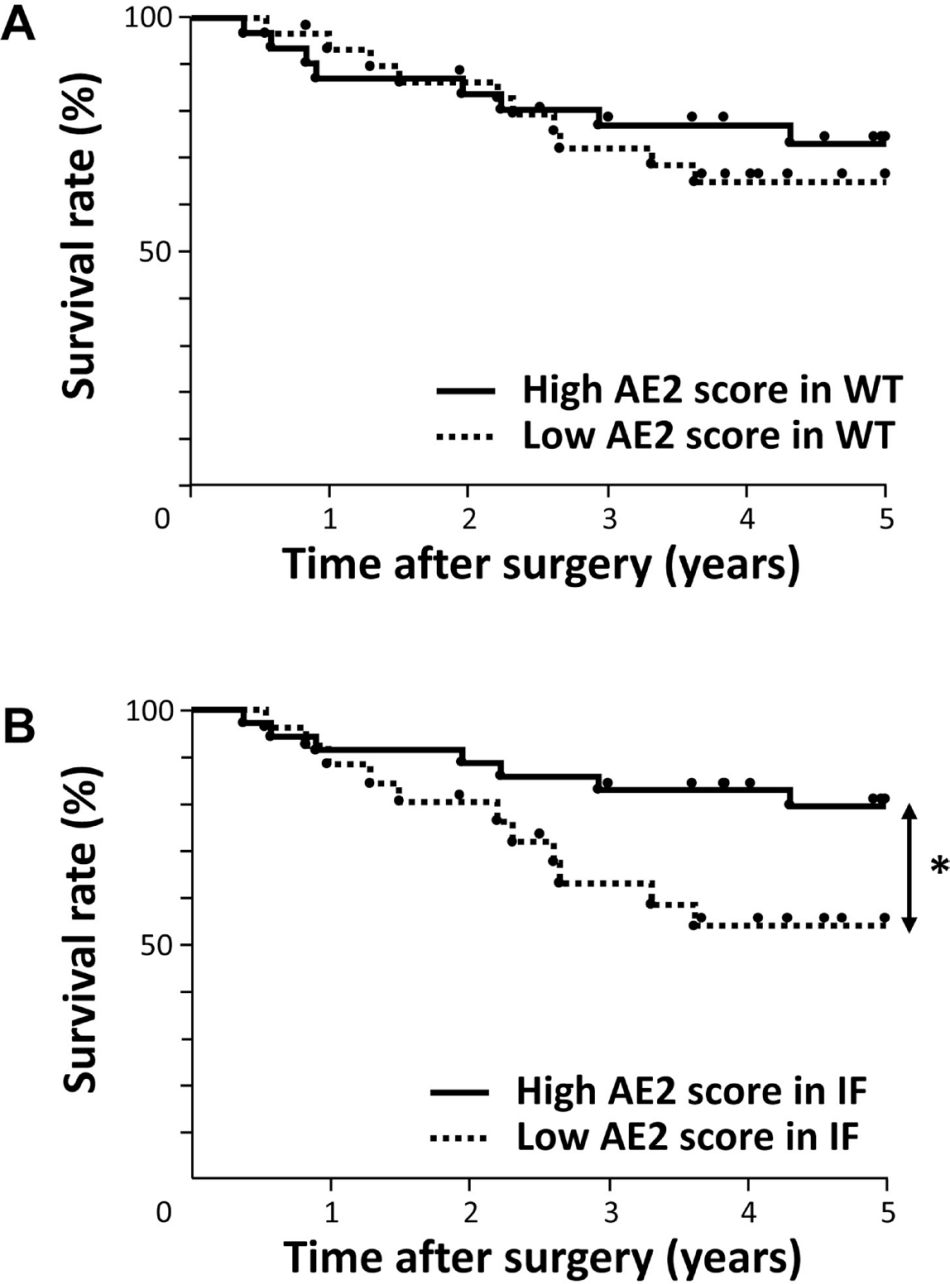
Survival curve of patients after curative resection for ESCC according to the expression of AE2 (**A**) Patients were classified into two groups: low-grade expression of AE2 (*n* = 30) and high-grade expression of AE2 (*n* = 31) in the whole tumor (WT). (**B**) Patients were classified into two groups: low-grade expression of AE2 (*n* = 26) and high-grade expression of AE2 (*n* = 35) at the invasive front of the tumor (IF). ^*^p < 0.05: Log-rank test.

We assessed the prognostic impact of the expression of AE2 for patients with ESCC. Regarding the expression of AE2 in WT, no significant difference was observed in the overall 5-year survival rate between patients with the low-grade expression of AE2 and those with the high-grade expression of AE2 in WT (Figure 5A, Table 3). Regarding the expression of AE2 at the IF, the 5-year overall survival rate of patients with the low-grade expression of AE2 (53.9%) was significantly lower than that of patients with the high-grade expression of AE2 in IF (79.4%) (*p* = 0.0388) (Figure 5B, Table 3).

**Table 3:** Prognostic factors of ESCC according to univariate and multivariate analyses

Variable	Univariate		Risk ratio	Multivariate	
	5-year survival rate (%)	*p* value		95% CI	*p* value
Gender					
Male	67.60	0.5737			
Female	76.19				
Age					
<65 years	65.91	0.6607			
≥65 years	73.23				
Tumor size					
<50 mm	68.36	0.7706			
≥50 mm	70.83				
Histological type					
Well-/moderately differentiated SCC	70.91	0.5023			
Poorly differentiated SCC	63.73				
Lymphatic invasion					
Negative	77.04	0.1597			
Positive	61.66				
Venous invasion					
Negative	78.77	0.0349^*^	Ref		0.0127^#^
Positive	55.38		3.9366	1.3404–12.3847	
pT					
pT1	84.21	0.0107^*^	Ref		0.2041
pT2-3	56.72		2.4893	0.6312–13.6307	
pN					
pN0	80.89	0.0944	Ref		0.1658
pN1-3	59.65		2.3638	0.7141–9.8557	
pStage					
pStage 0–I	87.50	0.0607	Ref		0.6366
pStage II–IV	61.99		1.7698	0.1486–18.3995	
AE2 staining score in WT					
High	73.19	0.5416			
Low	64.97				
AE2 staining score at the IF					
High	79.40	0.0388^*^	Ref		0.0326^#^
Low	53.86		3.2117	1.1014–9.8567	

SCC: squamous cell carcinoma; pT: pathological T stage; pN: pathological N stage; pStage: pathological stage; Ref: referent.

^*^p < 0.05: Log-rank test.

^#^*p* < 0.05: Cox's proportional hazards model; 95% CI: 95% confidence interval.

We also investigated which of the 11 variables studied (gender, age, tumor size, histological type, lymphatic invasion, venous invasion, pT and pN categories, pStage, AE2 expression in WT, and AE2 expression at the IF) influenced overall survival following the curative resection of esophageal cancer. A univariate analysis of overall survival after esophagectomy revealed that venous invasion, the pT category, and AE2 expression at the IF were significant prognostic factors (*p* = 0.0349, 0.0107, and 0.0388, respectively) (Table 3). A multivariate analysis with variables whose *P* values were less than 0.100 in the univariate analysis identified venous invasion and the AE2 staining score at the IF as independent prognostic factors (*p* = 0.0127 and 0.0326, respectively) (Table 3), suggesting that the weak expression of AE2 at the IF is a valuable poor prognostic factor for patients with ESCC. The results of immunohistochemistry with ESCC tissues were consistent with those obtained in our *in vitro* studies, and indicated the critical role of AE2 in the regulation of cellular movement of ESCC.

## DISCUSSION

AE2 is expressed in numerous cell types, is involved in the control of transepithelial ion transport and maintenance of pHi, and drives a cellular housekeeping function [14, 20, 27]. In the gastrointestinal tract, AE2 is distributed in the basolateral membrane of parietal cells and serves tissue-specific functions, such as acid secretion. A previous *in vivo* study showed that AE2−/− mice were emaciated, toothless, and exhibited severe growth retardation, and most died around the time of weaning, suggesting its critical significance in life support [27]. In esophageal tissue, AE2 contributes to HCO_3_^−^ secretion in esophageal submucosal glands [28, 29].

Although several recent studies described important roles for AE2 in various cancers [18–26], a consensus has not yet been reached about its expression and prognostic significance. In gastric cancer, Yang *et al.* showed that AE2 was down-regulated in cancerous gastric body specimens using immunohistochemical staining [18]. Wang *et al.* reported that AE2 expression was decreased in gastric cancer tissue, and these decreased levels correlated with the poor differentiation and prognosis of gastric cancer [20]. On the other hand, Song *et al.* revealed using immunohistochemistry that the AE2 protein was more strongly expressed in colon cancerous tissue than in adjacent non-tumor tissue, and that its expression correlated with a poor prognosis in patients with colon cancer [24]. Wu *et al.* showed that AE2 levels were significantly higher in human hepatocellular carcinoma tissue than in adjacent normal areas [21]. However, the clinical significance of AE2 expressed in human ESCC has not yet been examined. Our results from immunohistochemical staining revealed that the weak expression of AE2 at the IF correlated with the poor prognosis of patients with ESCC, supporting previous conclusions with gastric cancer [18, 20]. Possibly, cancer cells may require hypoxia-inducible AE2 distribution. Several reports including our previous study demonstrated that the expression of hypoxia-inducible factors was elevated with the change of pH regulators, such as carbonic anhydrase (CA), suggesting that they become effective prognostic factors in microenvironment where the expression of hypoxia-inducible factors is increased [11, 13, 30]. Although this mechanism needs to be elucidated in more detail in further studies, our results suggest the important and specific roles of AE2 at the IF of cancer. On the other hand, we need to state the limitation of this retrospective study related to small sample size and selection bias because our eligibility criteria was no preoperative chemotherapy. In fact, preoperative therapy has been performed positively for advanced ESCC in Japan [31]. However, to the best of our knowledge, this is the first study to investigate the expression of AE2 in human ESCC samples and perform gene expression profiling. The results obtained suggest a role for AE2 in cellular movement as well as the importance of its distribution in tumors as a prognostic predictor.

Regarding the mechanisms by which AE2 plays a role in tumor progression, previous studies demonstrated the regulation of pHi and/or pHe driven by a series of ion transporters [5, 6, 20]. Cancer cells have more alkaline pHi and acidic pHe values than normal cells, and the maintenance of intracellular alkalinization plays a critical role in the active progression of cancer including cell migration [6]. Under normal physiological conditions, AE family proteins are generally activated by intracellular alkalosis, and contribute to a decrease in pHi [5]. Our *in vitro* studies with ESCC cells revealed that the depletion of AE2 using siRNA increased cellular movement and decreased apoptosis, suggesting that the activity of cancer processes was driven by the maintenance of intracellular alkalinization. Wang *et al.* also showed that the overexpression of AE2 decreased pHi and this was accompanied by decreased cell growth and the migration of gastric cancer cells, supporting the mechanism via pHi [20]. Furthermore, the pH gradient across the cell membrane created by alkaline pHi and acidic pHe is known to increase tumor progression. Previous studies reported that acidic microenvironments affected the expression of some genes, such as MMP-9 [32, 33]. The results of the microarrays performed in the present study also revealed that the gene expression of important factors in cellular movement, such as MMP1, MMP12, and TIMP4, metalloproteinase inhibitor, was changed by the knockdown of AE2, suggesting that AE2 regulates the tumor behavior of ESCC via this pathway. On the other hand, our data showed that membrane protein AE2 was also localized in the cytoplasm of ESCC cells, and the similar phenomenon has been reported in other ion transporters. For instance, sodium iodide symporter is localized in the cytoplasm and does not mediate Na^+^/I^−^ transport in many carcinomas. Regarding the mechanism on carcinogenesis, it activates major signaling pathways via interaction with transcription factors or other molecules [34, 35]. Similarly, further studies may clarify important roles for AE2 in oncogenesis, independently of its transmembrane transport activity.

Regarding the other isoforms of AE in ESCC, we previously investigated the roles of AE1, which is known to be specifically expressed on the surface of erythrocytes [11]. Our findings showed that the depletion of AE1 using siRNA inhibited cell proliferation, migration, and invasion and induced apoptosis in ESCC cells. Furthermore, immunohistochemistry revealed that AE1 was overexpressed in ESCC tissue, and that diffuse AE1 expression was a valid poor prognostic factor for advanced esophageal cancer, suggesting that the role of AE1 in the regulation of tumor behavior is opposite to that of AE2 [11]. Interestingly, a similar phenomenon has been reported in gastric cancer cells [20]. Wang *et al.* showed that AE2 expression negatively correlated with AE1 expression in gastric cancer tissues [20]. Furthermore, low levels of AE2 and high levels of AE1 correlated with the poor survival of gastric cancer patients. Similarly, our data showed that ESCC patients with diffuse AE1expression and low levels of AE2 at the IF had the poorest survival (Supplementary Figure 11). On the other hand, ESCC patients with focal AE1expression and high levels of AE2 at the IF showed the highest 5-year overall survival rate (Supplementary Figure 11). Regarding this mechanism, they reported that an interaction between AE1 and p16 was a key event in gastric cancer progression and also that AE1/p16 expression promoted AE2 degradation [20]. Further investigations may reveal a similar regulatory mechanism between AE1 and AE2 in ESCC cells.

In summary, we herein demonstrated that AE2 played a role in the movement and survival of ESCC cells. The results of microarrays revealed that AE2 strongly affected the expression of genes related to MMPs and metalloproteinase inhibitors. The results of our immunohistochemical analysis also suggest that the decreased expression of AE2 is a valuable poor prognostic indicator in patients with ESCC. Although further investigations are needed, the results of the present study indicate that AE2 has potential as an important biomarker for tumor development and/or a novel therapeutic target for ESCC in the future.

## MATERIALS AND METHODS

### Cell lines, antibodies, and other reagents

The human ESCC cell lines TE2, TE5, TE9, and TE13 were obtained from the Riken Cell Bank (Tsukuba, Japan). The human ESCC cell line KYSE170 was obtained from the Japanese Collection of Research Bioresources Cell Bank (Osaka, Japan). These cells were grown in RPMI-1640 medium (Nacalai Tesque, Kyoto, Japan) supplemented with 100 U/ml of penicillin, 100 μg/ml of streptomycin, and 10% fetal bovine serum (FBS). Cells were cultured in flasks or dishes in a humidified incubator at 37°C under 5% CO_2_ in air.

The monoclonal anti-AE2 antibody used in the immunohistochemical analysis was obtained from Novus Biologicals (Littleton, CO). The monoclonal anti-AE2 antibody used in the protein assay was obtained from Santa Cruz Biotechnology (Dallas, TX). Rabbit polyclonal antibodies against GAPDH were purchased from Santa Cruz Biotechnology (Santa Cruz, CA). Horseradish peroxidase (HRP)-conjugated anti-rabbit or mouse secondary antibodies were purchased from Cell Signaling Technology (Beverly, MA).

### Western blotting

Cells were harvested in M-PER lysis buffer (Pierce, Rockford, IL) supplemented with protease inhibitors (Pierce). Protein concentrations were measured with a modified Bradford assay (Bio-Rad, Hercules, CA). Cell lysates containing equal amounts of total protein were separated by SDS-PAGE and then transferred onto PVDF membranes (GE Healthcare, Piscataway, NJ). These membranes were then probed with the indicated antibodies, and proteins were detected using an ECL Plus Western Blotting Detection System (GE Healthcare).

### Immunofluorescent staining

KYSE170 cells were cultured on glass coverslips and fixed with 4% paraformaldehyde, permeabilized with 0.1% Triton X-100, blocked with 1% BSA, and stained with the designated antibodies and rhodamine phalloidin. Slides were mounted with VECTASHIELD mounting medium and 4′,6-diamidino-2-phenylindole (DAPI) (Vector Laboratories, Burlingame, CA), and the distribution of AE2 was examined by fluorescence microscopy (BZX710; Keyence, Osaka, Japan).

### siRNA transfection

Cells were transfected with 12 nmol/l AE2 siRNA (Stealth RNAi™ siRNA #HSS109833; Invitrogen, Carlsbad, CA) using the Lipofectamine RNAiMAX reagent (Invitrogen) according to the manufacturer's instructions. Medium containing siRNA was replaced with fresh medium after 24 h. The control siRNA provided (Stealth RNAi™ siRNA Negative Control; Invitrogen) was used as a negative control.

### Overexpression study

Control-HaloTag^®^ plasmid (Promega, G6591) and AE2-HaloTag^®^ plasmid were transfected into KYSE170 cells using FuGENE HD transfection reagents (Promega, E2311) following the manufacturer's instructions. Transfection of vector was confirmed by fluorescent microscopy for HaloTag^®^ fusion protein stained with the TMR conjugated HaloTag^®^ ligand (Promega, G8252) according to the manufacturer's protocol. After passaging cells, AE2-expressing cells were used for proliferation and migration assays.

### Cell proliferation

Cells were seeded on 6-well plates at a density of 1.0 × 10^5^ cells per well and incubated at 37°C with 5% CO_2_. siRNA was transfected 24 h after the cells had been seeded. Cells were detached from the flasks with trypsin-EDTA 24, 48, and 72 h after siRNA transfection and were counted using a hemocytometer.

### Cell cycle analysis

The cell cycle phase was evaluated 48 h after transfection by flow cytometry. Briefly, cells were detached from the flasks by a trypsin-EDTA treatment, and nuclear isolation medium (NIM-DAPI 10, Beckman Coulter, Fullerton, CA, USA) was added to the cell pellets in order to stain cells. At least 10000 cells were analyzed using Cell Lab Quanta (Beckman Coulter), and FlowJo software was used to assess cell cycle distribution.” (Materials and Method part, page 13).

### Xenograft models in nude mice

Suspensions of 2.0 × 10^5^ AE2-HaloTag^®^ plasmid transfected KYSE170 cells in 100 μL of Matrigel^®^ Basement Membrane Matrix (Corning, NY) with PBS were injected subcutaneously into one side of the lower flanks of 4-week-old female nude mice, and the same amount of Control-HaloTag^®^ plasmid transfected cells were injected into the opposite side. At 21 days after the injection, all mice were sacrificed, and the weights and volumes of resected tumors were measured. Tumor volumes were calculated using the formula [36]:

Tumor volume = length × width^2^ × 0.5.

### Analysis of apoptotic cells

Cells were harvested 48 h after siRNA transfection and stained with fluorescein isothiocyanate-conjugated annexin V and phosphatidylinositol using the annexin V kit (Beckman Coulter, Brea, CA) according to the manufacturer's protocol. The proportion of apoptotic cells was analyzed by flow cytometry with BD Accuri C6 (BD Biosciences).

### Analysis of cell migration and invasion

The migration assay was conducted using a Cell Culture Insert with a pore size of 8 μm (BD Biosciences). Biocoat Matrigel (BD Biosciences) was used to evaluate cell invasion potential. Briefly, cells (1.0 × 10^5^ cells per well) were seeded in the upper chamber in serum-free medium 24 h after siRNA transfection. The lower chamber contained medium with 10% FBS. The chambers were incubated at 37°C for 48 h in 5% CO_2_, and non-migrated or non-invaded cells were then removed from the upper side of the membrane by scrubbing with cotton swabs. Migrated or invaded cells were fixed on the membrane and stained with Diff-Quick staining reagents (Sysmex, Kobe, Japan). The migrated or invaded cells on the lower side of the membrane were counted in four independent fields of view at 100× magnification for each insert. Each assay was performed in triplicate.

### Wound healing assay

AE2 siRNA- and control siRNA-transfected cells were re-seeded to each plate. Wounds were created in confluent cells using a pipette tip. The cells were then rinsed with medium to remove floating cells and debris. Culture plates were incubated at 37°C. Wounds were measured at 0, 6, 12, and 18 h. Cell-free areas were calculated using ImageJ software (http://rsb.info.nih.gov/ij/). Assays were repeated four times for each condition.

### Real-time reverse transcription-polymerase chain reaction (RT-PCR)

Total RNA was extracted using an RNeasy kit (Qiagen, Valencia, CA). Messenger RNA (mRNA) expression was measured by quantitative real-time PCR (7300 Real-Time PCR System; Applied Biosystems, Foster City, CA) with TaqMan Gene Expression Assays (Applied Biosystems), according to the manufacturer's instructions. The expression levels of the following genes were measured: AE2 (Hs01586776_m1), MMP1 (Hs00899658_m1), MMP12 (Hs00159178_m1), and TIMP4 (Hs00162784_m1) (Applied Biosystems). Expression was normalized for each gene to the housekeeping gene beta-actin (ACTB, Hs01060665_g1; Applied Biosystems). Assays were performed in triplicate.

### Microarray sample preparation and hybridization

Total RNA was extracted using an RNeasy kit (Qiagen). RNA quality was monitored with an Agilent 2100 Bioanalyzer (Agilent Technologies, Santa Clara, CA). Cyanine-3 (Cy3)-labeled cRNA was prepared from 0.1 μg of total RNA using a Low Input Quick Amp Labeling Kit (Agilent), according to the manufacturer's instructions. Samples were purified using RNeasy columns (Qiagen). A total of 0.60 μg of Cy3-labeled cRNA was fragmented and hybridized to an Agilent SurePrint G3 Human Gene Expression 8 × 60 K Microarray for 17 h. Slides were washed and scanned immediately on an Agilent DNA Microarray Scanner (G2565CA) using the one-color scan setting for 8 × 60 K array slides.

### Processing of microarray data

Scanned images were analyzed with Feature Extraction Software 10.10 (Agilent) using default parameters to obtain background-subtracted and spatially detrended Processed Signal intensities. Signal transduction networks were analyzed using Ingenuity Pathway Analysis (IPA) software (Ingenuity Systems, Inc., Redwood City, CA).

### Patients and primary tissue samples

ESCC tumor samples were obtained from 61 patients with histologically confirmed primary ESCC who underwent esophagectomy at Kyoto Prefectural University of Medicine between 1999 and 2009 and were embedded in paraffin after 12 h of formalin fixation. Patient eligibility criteria were as follows: no synchronous or metachronous cancers (in addition to ESCC) and no preoperative chemotherapy or radiation therapy. We excluded patients with non-curative resected tumors or non-consecutive data. All patients provided written informed consent. Relevant clinicopathological and survival data were obtained from the hospital database. Twenty patients (32.8%) died of cancer recurrence, and no patients died of other disease. The median follow-up period of all patients was 56.2 months (range, 4–157 months). Staging was principally based on the International Union Against Cancer (UICC)/TNM Classification of Malignant Tumors (7th edition) [37].

### Immunohistochemistry

Paraffin sections (thickness of 4 μm) of tumor tissues were subjected to immunohistochemical staining for the AE2 protein using the avidin-biotin-peroxidase method. Briefly, paraffin sections were dewaxed with xylene and hydrated with a graded series of alcohols. Endogenous peroxidases were quenched by incubating the sections for 30 min in 0.3% H_2_O_2_. An Avidin/Biotin Blocking Kit was used to block endogenous biotin, biotin receptors, and avidin binding sites (Vector laboratories, Burlingame, CA). Sections were then treated with a protein blocker and incubated at 4°C overnight with the anti-AE2 antibody. The avidin-biotin-peroxidase complex (Vectastain ABC Elite kit; Vector Laboratories, Burlingame, CA) was visualized with diaminobenzidine tetrahydrochloride. Sections were counterstained with hematoxylin, dehydrated with a graded series of alcohols, cleared in xylene, and mounted.

Immunohistochemical samples stained with AE2 were graded semi-quantitatively by considering both the staining intensity and percentage of positive tumor cells using an immunoreactive score (IRS) [38]. Staining intensity was scored as 0 (no staining), 1 (weak staining), 2 (moderate staining), or 3 (strong staining). The proportion of positive tumor cells was scored from 0 to 1.0. The score of each sample was calculated as the maximum multiplied product of the intensity and proportion scores (0 to 3.0).

### Statistical analysis

Fisher's exact test was used to assess differences between proportions, and Student's *t*-tests (for comparisons between two groups) were used to evaluate continuous variables. Overall survival curves were constructed by the Kaplan-Meier method, and differences in survival were examined using the Log-rank test. A multivariate analysis of the factors influencing survival was performed using the Cox proportional hazard model. Differences were considered significant when the relevant *P* value was < 0.05. These analyses were performed using the statistical software JMP (version 10, SAS Institute Inc., Cary, NC). Correlation analyses were performed by creating Fit *Y* by *X* plots using JMP.

## SUPPLEMENTARY MATERIALS FIGURES AND TABLES


