# Supplementary material for: Anion exchanger 2 suppresses cellular movement and has prognostic significance in esophageal squamous cell carcinoma
Source: Oncotarget. 2018 May 25;9(40):25993–6006. doi: 10.18632/oncotarget.25417 (PMC5995252; doi:10.18632/oncotarget.25417)
Supplement: Supplementary file 1 [file oncotarget-09-25993-s001.pdf]

Anion exchanger 2 suppresses cellular movement and has prognostic significance in esophageal squamous cell carcinoma

SUPPLEMENTARY MATERIALS

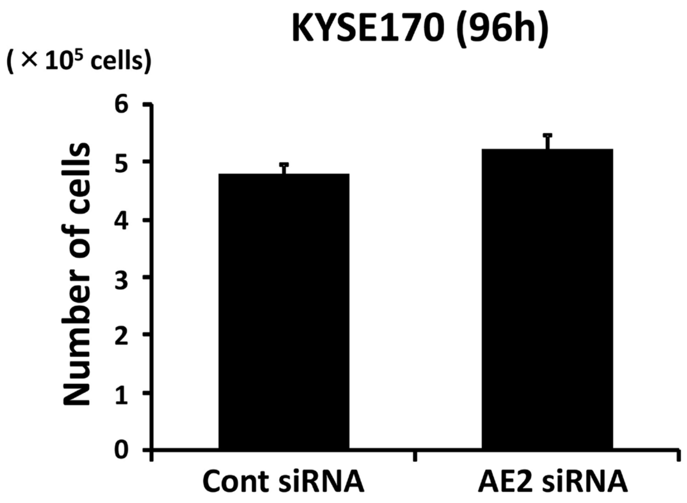

Supplementary Figure 1: The down-regulation of AE2 did not change the proliferation of KYSE170 cells. The number of cells was counted 96 h after siRNA transfection. Mean ± SEM. $n = 4$. $^{*}p < 0.05$ (significantly different from control siRNA).

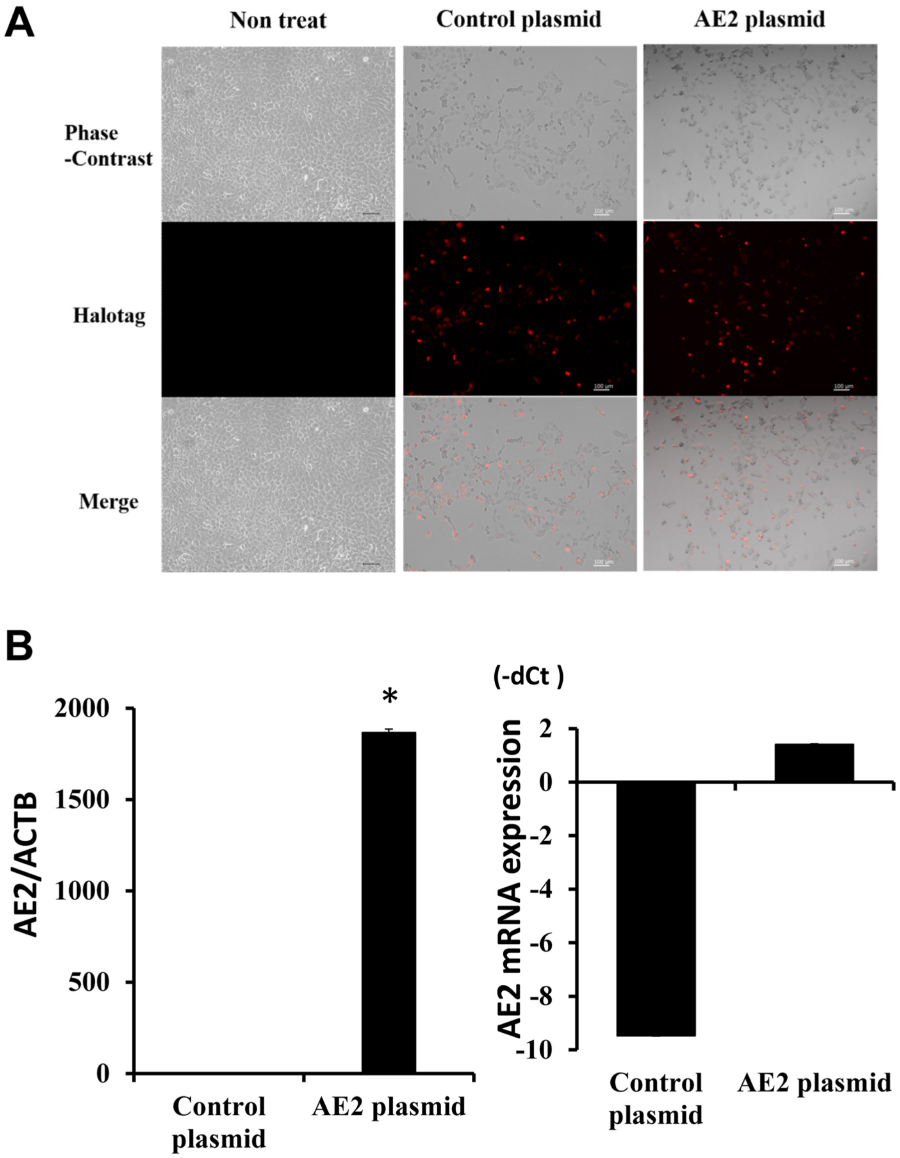

Supplementary Figure 2: AE2 overexpression in ESCC cells. (**A**) Fluorescent microscopy for HaloTag® fusion protein. Cells transfected Control-HaloTag® plasmid and AE2-HaloTag® plasmid were stained in red. (**B**) AE2 plasmid increased AE2 mRNA levels in KYSE170 cells. Mean ± SEM. $n = 3$. $^{*}p < 0.05$ (significantly different from control plasmid).

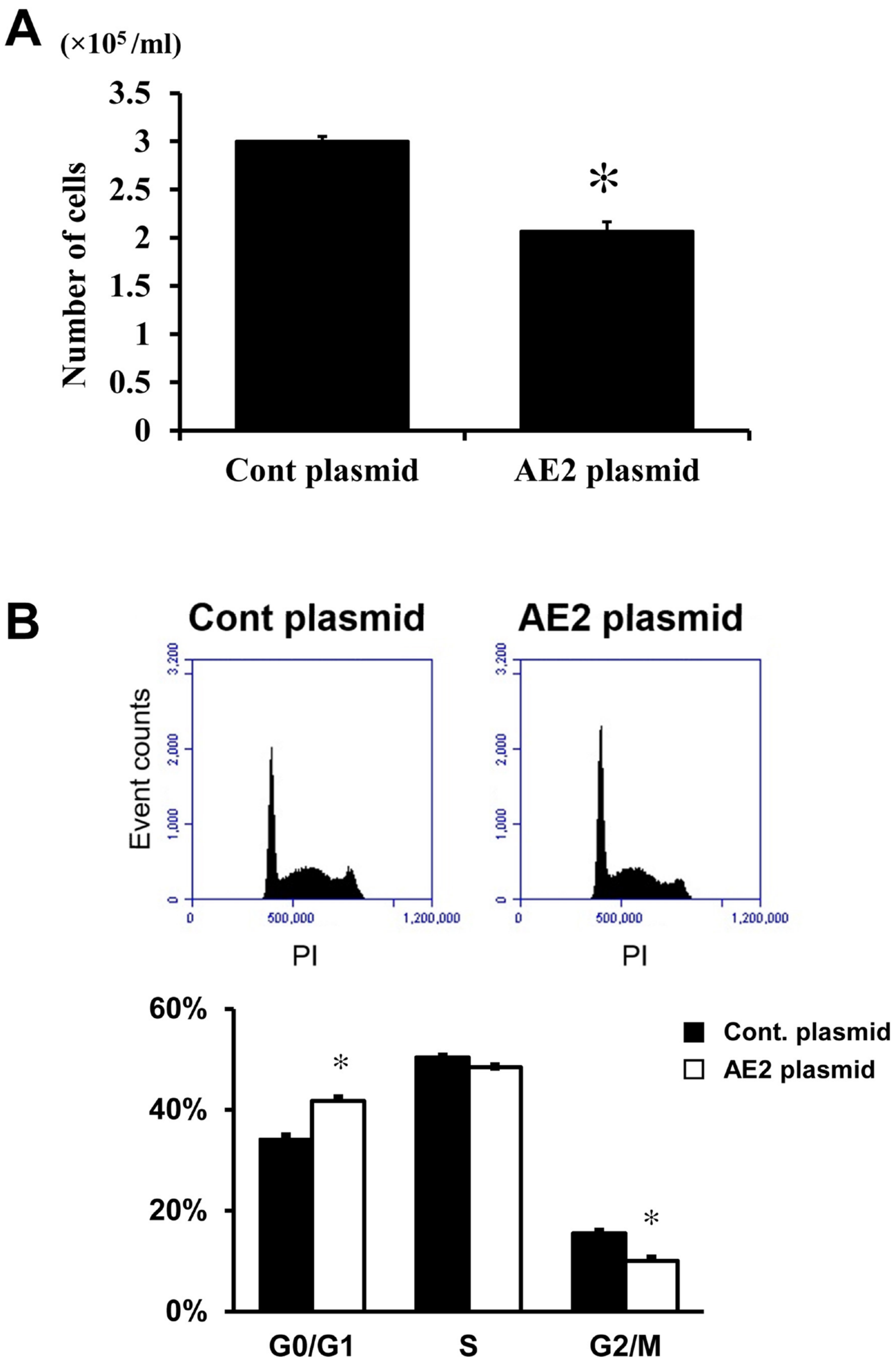

Supplementary Figure 3: AE2 overexpression decreased cell proliferation in ESCC cells. (**A**) The overexpression of AE2 decreased cell proliferation in KYSE170 cells. The number of cells was counted 48 h after plasmid transfection. Mean ± SEM. $n = 3$. $^{*}p < 0.05$ (significantly different from control plasmid). (**B**) AE2 overexpression partially reduced cell cycle progression from the G_1 to S phase in KYSE170 cells. Cells transfected Control-HaloTag® plasmid and AE2-HaloTag® plasmid were stained with propidium iodide (PI) and analyzed by flow cytometry. Mean ± SEM. $n = 3$. $^{*}p < 0.05$ (significantly different from control plasmid).

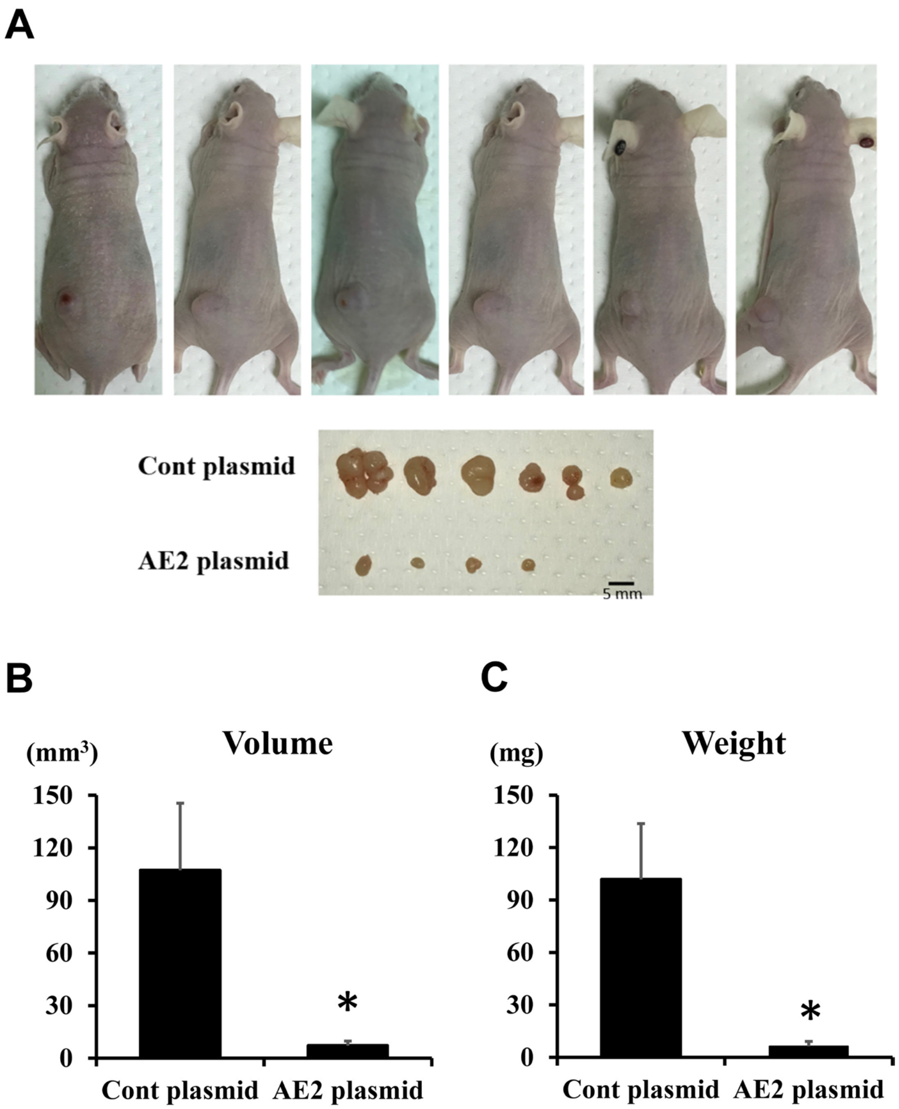

Supplementary Figure 4: Reduced tumor growth of AE2-HaloTag® plasmid transfected ESCC cells *in vivo*. Suspensions of 2.0×10^5 AE2-HaloTag® plasmid transfected KYSE170 cells in 100 µL of Matrigel® Basement Membrane Matrix with PBS were injected subcutaneously into right side of the lower flanks of 4-week-old female nude mice, and the same amount of Control-HaloTag® plasmid transfected cells were injected into the left side. (**A**) Photos of tumors from AE2 plasmid transfected KYSE170 cells in mice. AE2 plasmid transfected tumors were smaller than control tumors. (**B**) The volumes of resected tumors were significantly lower in AE2 plasmid transfected tumors in comparison with control tumors. Mean ± SEM. $n = 6$. $^*p < 0.05$ (significantly different from control plasmid). (**C**) The weights of resected tumors were significantly lower in AE2 plasmid transfected tumors in comparison with control tumors. Mean ± SEM. $n = 6$. $^*p < 0.05$ (significantly different from control plasmid).

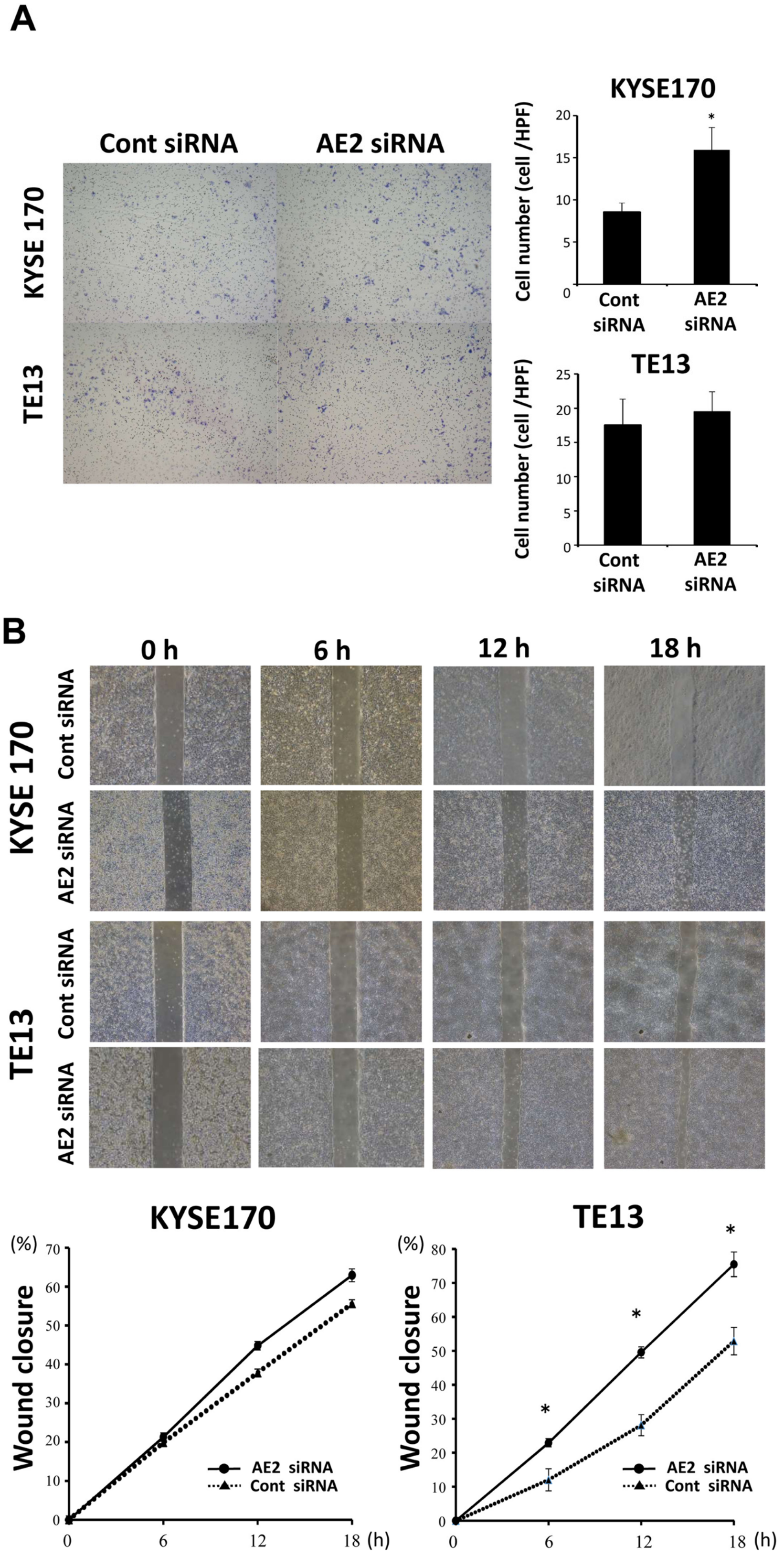

Supplementary Figure 5: AE2 controls the movement of ESCC cells. (**A**) The down-regulation of AE2 significantly increased cell invasion in KYSE170 cells. Cell invasion was examined using the Boyden chamber assay. Mean ± SEM. $n = 3$. $^{*}p < 0.05$ (significantly different from control siRNA). (**B**) The results of the wound healing assay were shown. The down-regulation of AE2 significantly increased wound closure in TE13 cells. Representative photomicrographs of the wounded cell monolayer are shown. The percentage of the cell-free area under each condition was calculated. Mean ± SEM. $n = 4$. $^{*}p < 0.05$ (significantly different from control siRNA).

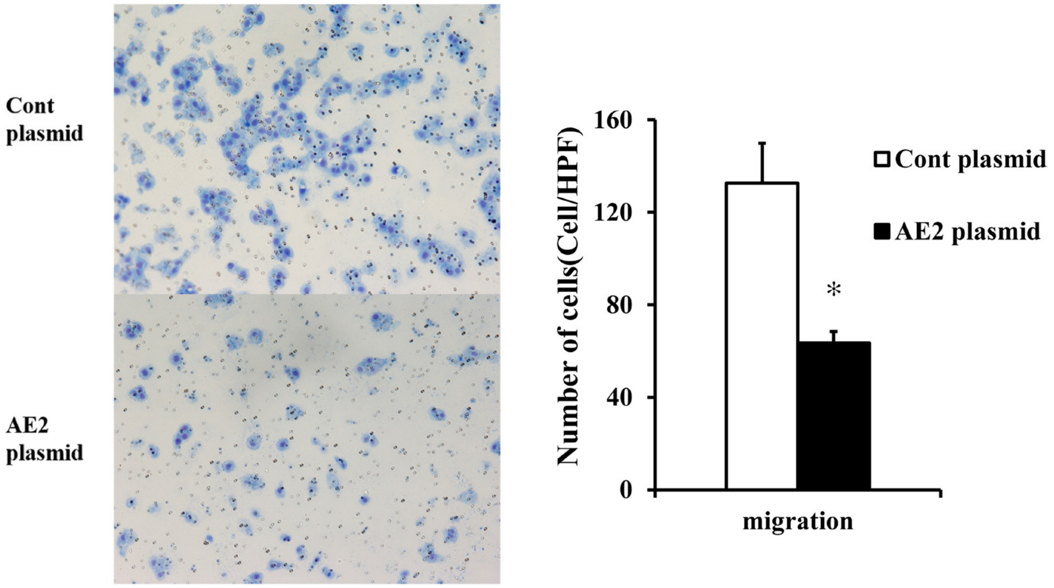

Supplementary Figure 6: AE2 overexpression decreased movement of ESCC cells. The overexpression of AE2 decreased cell migration in KYSE170 cells. Mean ± SEM. *n* = 3. $^{*}p < 0.05$ (significantly different from control plasmid).

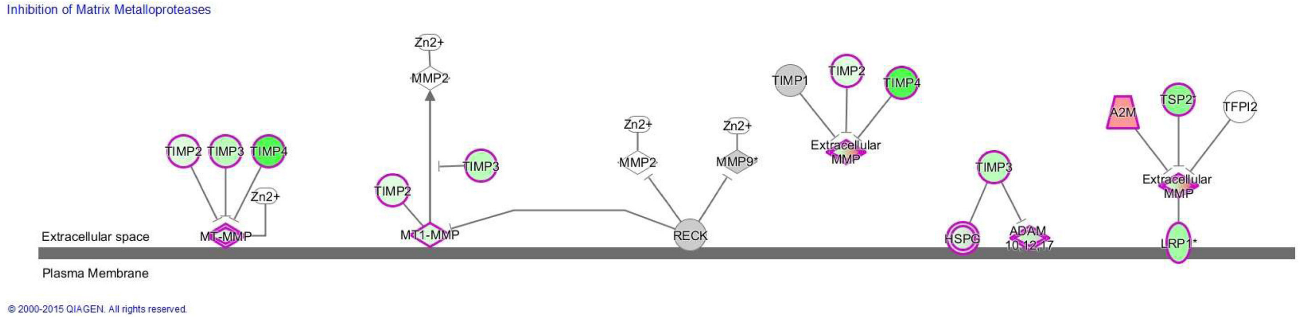

Supplementary Figure 7: The signaling map of "MMP" related to AE2 depletion according to an Ingenuity Pathway Analysis. Red and green indicate genes with expression levels that were higher or lower, respectively, than reference RNA levels.

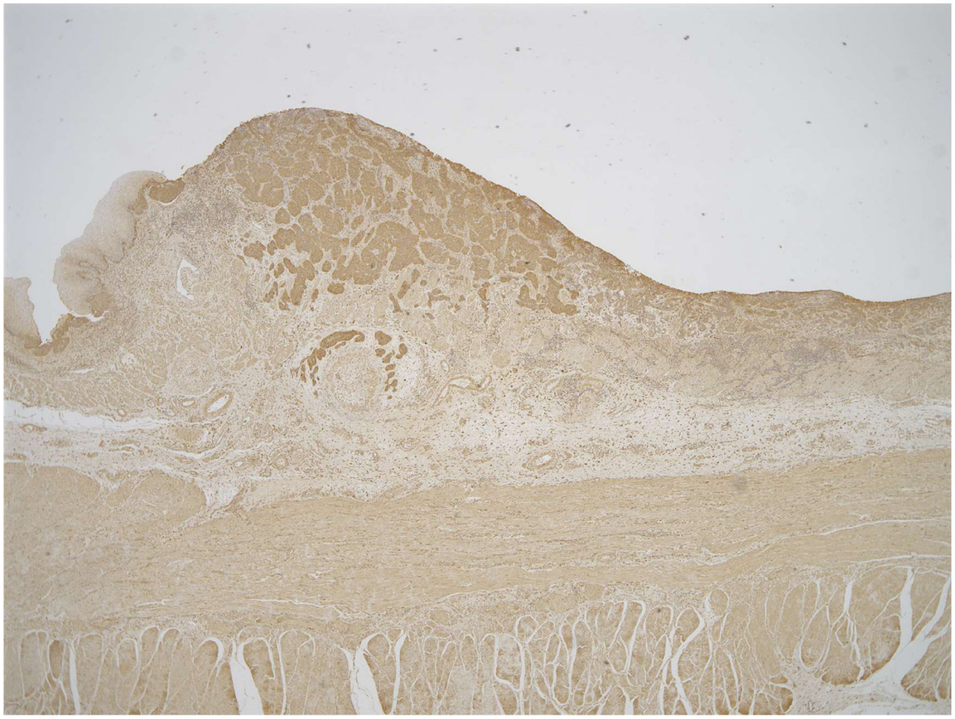

Supplementary Figure 8: AE2 protein expression in human ESCC. The sample includes both normal esophageal epithelia and ESCC cells.

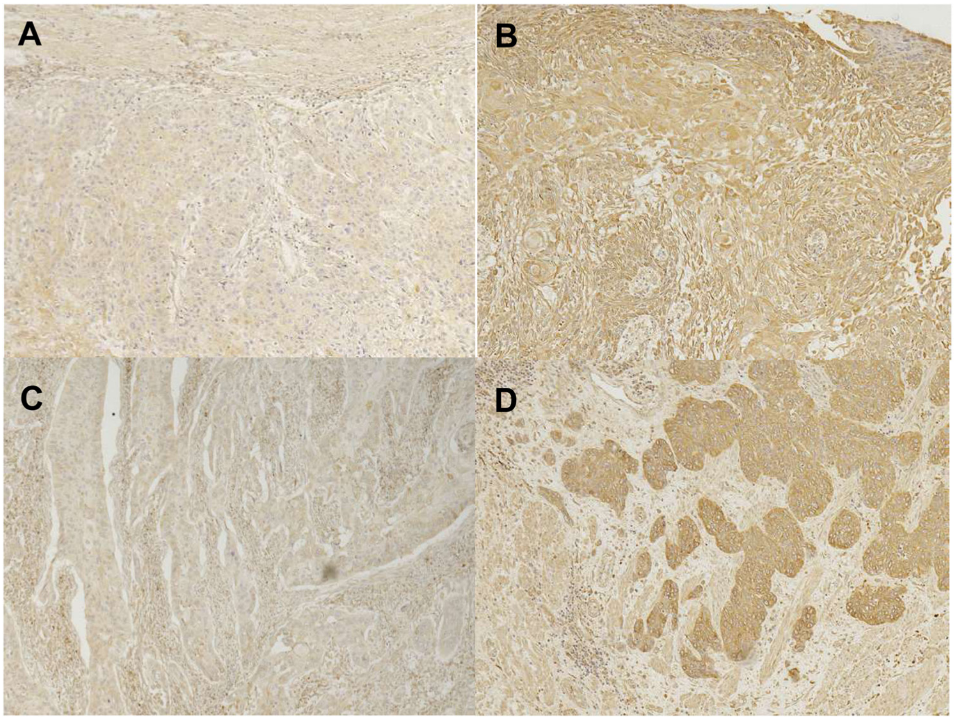

Supplementary Figure 9: AE2 protein expression in human ESCC. (**A**) Immunohistochemical staining of primary human ESCC samples with the low-grade expression of AE2 in the whole tumor (WT). Magnification: ×100. (**B**) Immunohistochemical staining of primary human ESCC samples with the high-grade expression of AE2 in WT. Magnification: ×100. (**C**) Immunohistochemical staining of primary human ESCC samples with the low-grade expression of AE2 at the invasive front of the tumor (IF). Magnification: ×100. (**D**) Immunohistochemical staining of primary human ESCC samples with the high-grade expression of AE2 at the IF. Magnification: ×100.

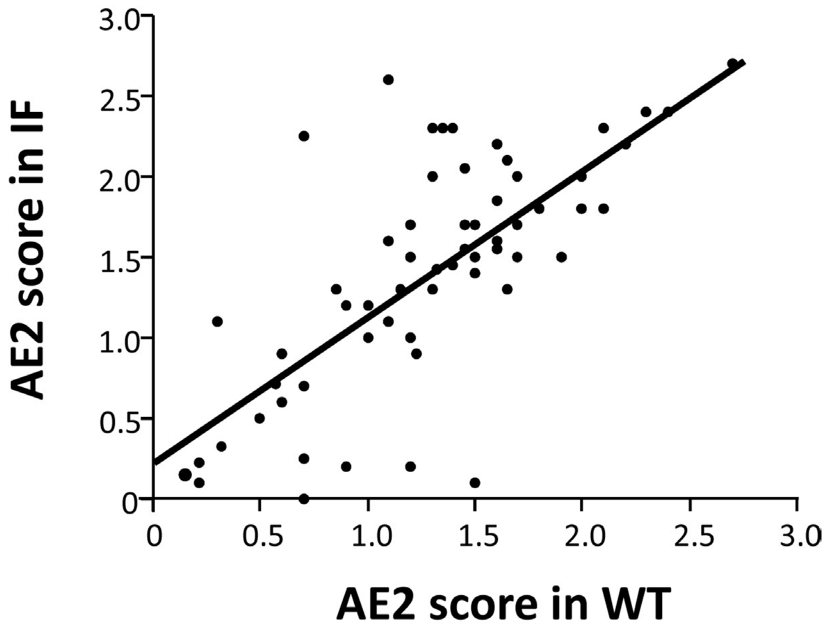

Supplementary Figure 10: A correlation analysis of the relationship between AE2 scores in the whole tumor (WT) and at the invasive front of the tumor (IF) was performed by producing Fit Y by X plots. The AE2 score in IF positively correlated with that in WT ($R^2 = 0.5319$, $p < 0.0001$).

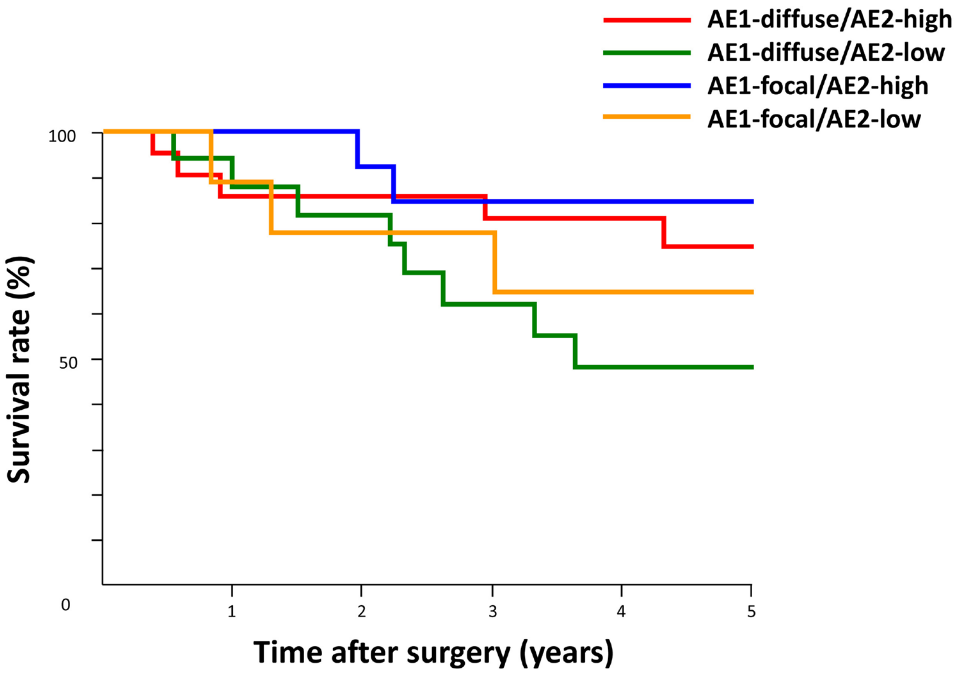

Supplementary Figure 11: Survival curve of patients after curative resection for ESCC according to the expression of AE1/AE2. Patients were classified into four groups: diffuse AE1 expression/high-grade expression of AE2 at the invasive front of the tumor (IF) ($n = 21$), diffuse AE1 expression/low-grade expression of AE2 at IF ($n = 17$), focal AE1 expression/high-grade expression of AE2 at IF ($n = 13$), focal AE1 expression/low-grade expression of AE2 at IF ($n = 9$).

Supplementary Table 1: Twenty genes displaying the greatest change in expression levels in AE2-depleted KYSE170 cells

Gene Symbol	Agilent ID	Gene Name	Exp Fold Change
**Up-regulated genes**			
FRMD7	A_32_P336776	FERM domain containing 7	146.626
KRTAP4-8	A_24_P325146	keratin-associated protein 4-8	110.507
SGCD	A_33_P3267380	sarcoglycan, delta (35kDa dystrophin-associated glycoprotein)	108.008
GADL1	A_33_P3244793	glutamate decarboxylase-like 1	76.321
C16orf86	A_32_P178499	chromosome 16 open reading frame 86	74.906
POLR2F	A_33_P3383561	polymerase (RNA) II (DNA directed) polypeptide F	63.294
C20orf203	A_32_P509677	chromosome 20 open reading frame 203	61.053
UCP1	A_23_P30091	uncoupling protein 1 (mitochondrial, proton carrier)	53.892
GAL3ST2	A_23_P326157	galactose-3-O-sulfotransferase 2	52.929
UPB1	A_23_P120822	ureidopropionase, beta	49.763
MTUS2	A_33_P3293858	microtubule-associated tumor suppressor candidate 2	47.373
LINC00954	A_21_P0001842	long intergenic non-protein coding RNA 954	46.981
SERPINI1	A_23_P166929	serpin peptidase inhibitor, clade I (neuroserpin), member 1	46.592
KLHL41	A_23_P17190	kelch-like family member 41	43.201
FAM107A	A_19_P00315587	family with sequence similarity 107, member A	40.420
PCDH10	A_33_P3294598	protocadherin 10	40.308
NOVA1	A_24_P270496	neuro-oncological ventral antigen 1	39.561
VNN1	A_33_P3399571	vanin 1	38.935
DNAH12	A_33_P3422010	dynein, axonemal, heavy chain 12	38.479
LOC102724193	A_21_P0006245	uncharacterized LOC102724193	38.055
**Down-regulated genes**			
**Gene Symbol**	**Agilent ID**	**Gene Name**	**Exp Fold Change**
PYGM	A_23_P405815	phosphorylase, glycogen, muscle	–264.294
OR2D2	A_33_P3264193	olfactory receptor, family 2, subfamily D, member 2	–226.914
SEC1P	A_33_P3548860	secretory blood group 1, pseudogene	–163.031
LOC100131472	A_21_P0005212	uncharacterized LOC100131472	–144.507
STARD9	A_23_P380010	StAR-related lipid transfer (START) domain containing 9	–128.178
LINC01330	A_21_P0002795	long intergenic non-protein coding RNA 1330	–117.458
HAVCR2	A_24_P411561	hepatitis A virus cellular receptor 2	–103.107
ANKRD6	A_33_P3278455	ankyrin repeat domain 6	–100.218
CISTR	A_21_P0007879	chondrogenesis-associated transcript	–98.224
NANOS2	A_33_P3388453	nanos homolog 2 (Drosophila)	–96.603
ST6GAL1	A_33_P3382276	ST6 beta-galactosamide alpha-2,6-sialyltranferase 1	–95.604
ASTN1	A_33_P3220738	astrotactin 1	–90.384
CCDC184	A_23_P344194	coiled-coil domain containing 184	–79.893
LOC255187	A_21_P0012962	uncharacterized LOC255187	–77.440
LOC728084	A_33_P3213665	uncharacterized LOC728084	–72.454
TAS2R19	A_23_P340308	taste receptor, type 2, member 19	–66.487
ALDH4A1	A_32_P192970	aldehyde dehydrogenase 4 family, member A1	–66.211
MAMLD1	A_33_P3404489	mastermind-like domain containing 1	–65.254
ABCB1	A_23_P82523	ATP-binding cassette, sub-family B (MDR/TAP), member 1	–64.579
CES1P1	A_23_P374892	carboxylesterase 1 pseudogene 1	–62.423

Supplementary Table 2: Top biological functions and canonical pathways of AE2 according to an ingenuity pathway analysis

**Top Biological Functions**		
**Diseases and Disorders**		
**Name**	***p* value**	**Number of Molecules**
Dermatological Diseases and Conditions	4.50E-03–1.90E-16	757
Cancer	5.17E-03–1.75E-09	1499
Organismal Injuries and Abnormalities	5.26E-03–1.75E-09	1507
Reproductive System Diseases	5.11E-03–1.75E-09	710
Immunological Diseases	4.07E-03–2.20E-09	271
**Molecular and Cellular Functions**		
**Name**	***p* value**	**Number of Molecules**
Cellular Movement	5.08E-03–2.86E-08	207
Cellular Development	5.37E-03–3.60E-07	243
Cellular Growth and Proliferation	5.43E-03–3.60E-07	293
Cell-to-Cell Signaling and Interaction	5.43E-03–5.24E-07	224
Cell Death and Survival	5.28E-03–9.57E-06	258
